# Caramel Color in Soft Drinks and Exposure to 4-Methylimidazole: A Quantitative Risk Assessment

**DOI:** 10.1371/journal.pone.0118138

**Published:** 2015-02-18

**Authors:** Tyler J. S. Smith, Julia A. Wolfson, Ding Jiao, Michael J. Crupain, Urvashi Rangan, Amir Sapkota, Sara N. Bleich, Keeve E. Nachman

**Affiliations:** 1 Johns Hopkins Center for a Livable Future, Johns Hopkins University, Baltimore, Maryland, United States of America; 2 Department of Environmental Health Sciences, Bloomberg School of Public Health, Johns Hopkins University, Baltimore, Maryland, United States of America; 3 Department of Health Policy and Management, Bloomberg School of Public Health, Johns Hopkins University, Baltimore, Maryland, United States of America; 4 Food Safety and Sustainability Center, Consumer Reports, Yonkers, New York, United States of America; 5 Maryland Institute for Applied Environmental Health, University of Maryland School of Public Health, College Park, Maryland, United States of America; 6 Risk Sciences and Public Policy Institute, Bloomberg School of Public Health, Johns Hopkins University, Baltimore, Maryland, United States of America; Vanderbilt University, UNITED STATES

## Abstract

Caramel color is added to many widely-consumed beverages as a colorant. Consumers of these beverages can be exposed to 4-methylimidazole (4-MEI), a potential carcinogen formed during its manufacture. California’s Proposition 65 law requires that beverages containing 4-MEI concentrations corresponding to exposures that pose excess cancer risks > 1 case per 100,000 exposed persons (29 μg 4-MEI/day) carry warning labels. Using ultrahigh-performance liquid chromatography-tandem mass spectrometry, we assessed 4-MEI concentrations in 12 beverages purchased in California and a geographically distant metropolitan area (New York) in which warning labels are not required. In addition, we characterized beverage consumption by age and race/ethnicity (using weighted means calculated from logistic regressions) and assessed 4-MEI exposure and resulting cancer risks and US population cancer burdens attributable to beverage consumption. Data on beverage consumption were obtained from the National Health and Nutrition Examination Survey, dose-response data for 4-MEI were obtained from the California Environmental Protection Agency Office of Environmental Health Hazards Assessment, and data on population characteristics were obtained from the U.S. Census Bureau. Of the 12 beverages, Malta Goya had the highest 4-MEI concentration (915.8 to 963.3μg/L), lifetime average daily dose (LADD - 8.04x10^-3^ mg/kgBW-day), lifetime excess cancer risk (1.93x10^-4^) and burden (5,011 cancer cases in the U.S. population over 70 years); Coca-Cola had the lowest value of each (4-MEI: 9.5 to 11.7μg/L; LADD: 1.01x10^-4^ mg/kgBW-day; risk: 1.92x10^-6^; and burden: 76 cases). 4-MEI concentrations varied considerably by soda and state/area of purchase, but were generally consistent across lots of the same beverage purchased in the same state/area. Routine consumption of certain beverages can result in 4-MEI exposures > 29 μg/day. State regulatory standards appear to have been effective in reducing exposure to carcinogens in some beverages. Federal regulation of 4-MEI in caramel color may be appropriate.

## Introduction

Soft drinks, including sodas, are widely consumed in the United States [1,2]. A common ingredient in many soft drinks (e.g., colas, root beers, iced teas) is caramel color [3,4] produced with ammonium compounds (i.e., caramel color type IV)[5,6]. The use of these compounds to manufacture caramel color can result in the formation of 4-methylimidazole (4-MEI)[7]. In recent years, evidence for the carcinogenicity of 4-MEI has raised concerns about uses of caramel color type III and IV that may expose consumers to 4-MEI and increase cancer risk [8–11].

The U.S. National Toxicology Program (NTP) assessed the carcinogenicity of 4-MEI in male and female mice and rats using two-year feeding studies in 2007, concluding that there was “clear evidence of carcinogenic activity in male and female mice”, based on increases in adenomas and carcinomas of the lung in exposed mice relative to controls, and “equivocal evidence of carcinogenic activity in female rats”, based on increases in leukemia in exposed female rats relative to controls [12]. No evidence of carcinogenicity was found in male rats [12]. Based on the NTP studies, the International Agency for Research on Cancer classified 4-MEI as Group 2B, or “possibly carcinogenic to humans”, indicating sufficient evidence of carcinogenicity in experimental animals while no human data were available [13].

In 2011, California listed 4-MEI as a carcinogen under the Safe Drinking Water and Toxic Enforcement Act of 1986, which is better known as Proposition 65 [14]. A product sold in California that contains a chemical listed as a carcinogen under Proposition 65 must carry a warning label if exposure to the chemical resulting from use of the product will exceed a no significant risk level (NSRL)[15]. The NSRL is the lifetime average daily exposure associated with a one-in-100,000 cancer risk [15], and is set at 29 μg/day for 4-MEI [14]. Following the listing of 4-MEI under Proposition 65, leading soft drink manufacturers announced that they would lower 4-MEI concentrations in products sold in the U.S.[9].

While the use of caramel color type III and IV continues to receive scrutiny [8,9], and studies of 4-MEI in foods and beverages have been reported [10,11,13], prior peer-reviewed studies have not assessed 4-MEI exposure or its associated cancer risk and burden. To address this critical gap in the literature, we conducted a market basket study of soft drinks that listed caramel color as an ingredient to estimate exposure to 4-MEI resulting from soft drink consumption, and estimated cancer risk and/or burden associated with exposure. The results were stratified by location to assess how Proposition 65 may have influenced 4-MEI exposure and risk. To facilitate the exposure assessment, we analyzed soft drink consumption using data from the National Health and Nutrition Examination Survey (NHANES).

## Materials and Methods

Our study did not involve human or animal subjects.

### Sample Collection and Preparation

We purchased one hundred and ten (110) soft drink samples in duplicate from retail stores in California and the New York metropolitan area (i.e., Connecticut, New Jersey, and New York). The two locations were chosen to include the jurisdiction where soft drinks are subject to Proposition 65 requirements (California) and a geographically distinct population center as a comparison area (the New York area), to allow for detection of differences in 4-MEI concentrations by location that may be attributable to Proposition 65. For each beverage/location combination (e.g., Diet Coke from California), at least two separate production lots were purchased to capture variability within that combination. All samples were purchased in can form, with the exception of Goya Malta, which was purchased in glass bottles.

The initial set of samples (n = 57) was purchased in April-May 2013. Two more sets of samples were purchased in July-September (n = 12) and December of 2013 (n = 29) to assess potential changes over time in 4-MEI concentrations. All samples were shipped to Johns Hopkins University for preparation for 4-MEI analyses. Twelve separate samples from these 98 lots were tested for 4-MeI levels a second time to ensure the accuracy of the initial testing results.

Samples were opened and poured into a 125mL amber glass jar or a 50mL clear polyethylene tube (Fisher Scientific, Hampton, New Hampshire). Each container was sealed with laboratory film (Parafilm, Bemis Company, Neenah, Wisconsin) and labeled with a randomly generated sample number to blind the contract laboratory to the identity of the soft drink during analysis. All samples were stored at room temperature from purchase through 4-MEI analysis.

### 4-MEI Analyses

Following preparation, beverage samples were sent to a commercial laboratory for analysis and analyzed for 4-MEI by ultrahigh-performance liquid chromatography-tandem mass spectrometry (UPLC-MS/MS), previously described by Wang and Schnute [16]. Details about the 4-MEI analyses are in the Supplemental Material. Twelve different beverage products were tested, including one that did not contain caramel color in its ingredient list (Sprite); this beverage did not have measurable concentrations of 4-MEI and was dropped from further analysis.

### NHANES Analyses

We used data from NHANES to estimate average daily consumption of different types of beverages. The NHANES is a population-based survey that uses a multi-stage, clustered, probability sampling strategy to collect information on health and nutrition in the U.S. population. Our analysis combined data from 2003–2010 to look at overall patterns during that time period. Our study sample included children and adults aged 3–70 years (unweighted N = 28,710). Survey respondents were excluded if their dietary recall was incomplete or unreliable (as determined by NHANES staff [17]) or if they were pregnant or had diabetes at the time of data collection. A complete description of data-collection procedures and analytic guidelines is available elsewhere (www.cdc.gov/nchs/nhanes.htm).

Survey respondents reported the type and quantity of all food and beverages consumed in the prior 24-hour period (midnight to midnight). All reported food and beverage items are then systematically coded using the U.S. Department of Agriculture (USDA) Food and Nutrient database. We analyzed consumption of all sodas, and further categorized soda into five mutually exclusive categories: 1) Cola, 2) Diet-Cola, 3) Root Beer, 4) Pepper Cola and 5) Other (non-diet) Cola. We examined beverage consumption overall and by age (3 to 6, 6 to <11, 11 to <16, 16 to <21, 21 to <45, 45 to <65, and 65 to 70). We also stratified beverage consumption by race (Non-Hispanic White, Non-Hispanic Black, Hispanic) among adults aged 21–70.

We used multivariate logistic regression models to estimate the percent of the population (aged 21 to 70) overall and stratified by race who consumed any of each beverage (Table A in S1 File). Multivariate linear regression (OLS) was then used to estimate the volume (mL) of each beverage consumed among drinkers. These models adjusted for sex, age (21 to 44, 45 to 64, 65 to 70), race/ethnicity (Non-Hispanic White, Non-Hispanic Black, Hispanic), education (< high school, high school or GED, > high school), employment status (employed, unemployed), income (low income ≤130% federal poverty level, high income), BMI (healthy, overweight, obese), and weight loss intention. We repeated this analysis stratified by age. Models for adults aged 21 to 70 adjusted for the above variables whereas models for children aged 3–20 adjusted for sex, race/ethnicity, income, and weight status. All covariates for both child and adult models were included based on prior literature regardless of statistical significance. Appropriate survey weights were used for all analyses to generate nationally-representative estimates.

Soft drinks included in the exposure and risk assessments were assigned to a corresponding NHANES beverage categories in order to estimate intake rates (IRs) and lifestage-specific consumption factors (CFs) (Table B in S1 File). A corresponding NHANES category was not available for malt beverages or iced tea, and medians of the median and 95^th^ percentile IRs and the CFs of the NHANES categories used for the exposure assessment (cola, diet cola, pepper cola, and root beer) were used for Malta Goya and Brisk Lemon Iced Tea.

### Exposure Assessment

We used the results of the 4-MEI and NHANES analyses to estimate lifetime average daily doses (LADD; milligrams per kilogram of bodyweight per day [mg/kgBW/day]) of 4-MEI associated with consumption of each beverage, accounting for several types of variability.

First, we estimated average daily dose (ADD; mg/kgBW/day) of 4-MEI for each lifestage *x → y*, using the formula:

$$ADDx\to y\;=\;\frac{\left[4MEI\right]\;x\;IRx\to y}{kgBWx\to y}/1,000,$$ (1)

where [4MEI] is the 4-MEI concentration (μg/L) (Table 1), IR is intake rate among consumers of the beverage (L/day) (Table 2; results were divided by 1,000 to convert mL/day to L/day), and kgBW is bodyweight (kg) (Table C in S1 File)[18]. The result was divided by 1,000 to yield dose in mg/kgBW/day.

**Table 1:** Mean and Maximum 4-MEI Concentrations (μg/L) in Beverage, by Brand, Beverage, and Location.

Brand	Model	Location	Mean	Maximum
365 Everyday Value	Dr. Snap Regular	All	61.1	163
365 Everyday Value	Dr. Snap Regular	CA	29.6^1^	30.8
365 Everyday Value	Dr. Snap Regular	NY	92.6^1^	163
A&W	Root Beer Regular	All	65.0	69.8
A&W	Root Beer Regular	CA	68.2	69.8
A&W	Root Beer Regular	NY	61.8	64.4
Brisk	Lemon Iced Tea	All	46.5	48.2
Brisk	Lemon Iced Tea	CA	46.0	48.2
Brisk	Lemon Iced Tea	NY	47.0	47.4
Coca Cola	Diet Coke	All	9.8	10.4
Coca Cola	Diet Coke	CA	9.5	10
Coca Cola	Diet Coke	NY	10.2	10.4
Coca Cola	Regular	All	11.7	12.6
Coca Cola	Regular	CA	12.1	12.6
Coca Cola	Regular	NY	11.3	11.7
Coca Cola	Zero	All	10.3	11.9
Coca Cola	Zero	CA	10.8	11.9
Coca Cola	Zero	NY	9.7	9.9
Dr. Pepper	Regular	All	28.0	30
Dr. Pepper	Regular	CA	28.4	30
Dr. Pepper	Regular	NY	27.5	28.8
Goya	Malta	All	945.5	1104
Goya	Malta	CA	963.3	1104
Goya	Malta	NY	915.8	1004
Pepsi	Diet	All	191.5	550
Pepsi	Diet	CA	78.4^1^	107
Pepsi	Diet	NY	304.5^1^	550
Pepsi	One	All	246.9	750
Pepsi	One	CA	119.7^2^	138
Pepsi	One	NY	501.5^2^	750
Pepsi	Regular	All	183.6	556
Pepsi	Regular	CA	75.9^1^	90
Pepsi	Regular	NY	291.2^1^	556
Sprite	Regular	All	<LOQ^3^	<LOQ
Sprite	Regular	CA	<LOQ	<LOQ
Sprite	Regular	NY	<LOQ	<LOQ

^1^Significant differences (p<0.05) and

^2^(p<0.001) in mean 4-MEI concentrations were observed between California and the New York metropolitan area samples.;

^3^LOQ = limit of quantitation

**Table 2:** Mean 4-MEI concentrations in beverages (in μg/L), by purchase location and 2013 month of sampling.

Beverage	Location	April/May	December
Diet Pepsi	California	75.4	70.9
Diet Pepsi	New York	514.7	94.3
Dr. Snap Regular	California	29.6	29.5
Dr. Snap Regular	New York	157.3	27.9
Goya Malta	California	842.7	890.5
Goya Malta	New York	965.3	866.3
Pepsi One	California	125.7	111.3
Pepsi One	New York	550.0	453.0
Pepsi Regular	California	69.8	81.9
Pepsi Regular	New York	491.3	91.1

For [4MEI], we used both the mean and maximum 4-MEI concentrations for samples purchased from all locations and from California and the New York area to account for variability among samples (the mean concentration compared to the maximum concentration) and location of purchase (the California samples compared to the New York area samples). For IR, we matched each beverage to an appropriate NHANES category (see below) and, for each lifestage *x→y*, used 50^th^- and 95^th^-percentile intake rates for that lifestage for that category, to account for variability in consumption across and within lifestages. For kgBW, we used the bodyweights recommended by the U.S. Environmental Protection Agency for each lifestage *x→y*[18], to account for variability in bodyweight across lifestages.

Second, we estimated LADD by time-weighting each ADD_x → y_ by the exposure duration (the duration of the lifestage) in years and averaging the sum of time-weighted ADDs over an averaging time, using the formula:

$$LADD\;=\;\sum_{i}^{n}(ADDx\;\to \;y\;x\;EDx\;\to \;y)\;/\;AT,$$ (2)

where ED_x → y_ is lifestage-specific exposure duration (Table C in S1 File) and AT is averaging time (70 years)[18].

### Risk Assessment

The cancer risk associated with consumption of each beverage was estimated by multiplying LADD by the unit cancer risk (UCR) for 4-MEI developed by the Office of Environmental Health Hazard Assessment (OEHHA) in California after the state listed 4-MEI as a carcinogen under Proposition 65:

Risk = LADD x UCR. (3)

The UCR is the increase in lifetime cancer risk per mg/kgBW/day unit dose. The OEHHA derived the UCR of 0.024 per mg 4-MEI/kgBW/day from data on the carcinogenicity of 4-MEI in the male mouse, as obtained from the NTP feeding study [12,14].

The cancer burden is the number of lifetime excess cancer cases associated with a population exposure to 4-MEI. In this case, we estimated the cancer burden associated with consumption of each beverage by the U.S. population, assuming that only one beverage is consumed nationally and that 4-MEI exposure does not occur from other sources. For the purpose of estimating burden, each ADD was adjusted by a lifestage-specific consumption factor, or the percentage of the U.S. population within the lifestage that consumes the beverage, as determined from the NHANES analyses, prior to calculation of the LADD:

$$LADD\;=\;\sum_{i}^{n}([ADDx\;\to \;y\;x\;CFx\;\to \;y]\;x\;EDx\;\to \;y)\;/\;AT,$$ (4)

where CF_x→y_ is the lifestage-specific consumption factor.

The adjusted LADD was then multiplied by the UCR to estimate cancer risk, as above. Finally, the cancer burden was estimated as:

Burden = Risk x P, (5)

where P is the U.S. population size at mid-year in 2012, as estimated by the U.S. Census Bureau [19].

## Results

### 4-MEI Analyses

Table 1 presents the mean and maximum 4-MEI concentrations (μg/L) in beverages, by brand, beverage, and location. There was wide variability in mean and maximum 4-MEI concentrations among the beverages, with the highest concentrations for all locations in Malta Goya (mean: 945.5μg/L; maximum: 1104μg/L) and the lowest concentrations for all locations in Diet Coke (mean: 9.8μg/L; maximum: 10.4μg/L) (Table 1). Details of quality control analyses are in S1 File.

For most combinations of beverages and locations, there was little variability between the mean and maximum 4-MEI concentrations (< 20% variation for 19 out of 24 beverage and location combinations). More notable variability was observed in Dr. Snap Regular (76% variability between mean and maximum New York area samples) and the three Pepsi beverages (50–90% for New York area samples, depending on the beverage, and 36% for California samples of Diet Pepsi). When results for the five combinations with variability > 20% are stratified by month of purchase, however, variability in 4-MEI concentrations declined markedly within beverage over time. For example, in Diet Pepsi samples from California, variability declined from 25% in samples purchased between April and July to 7% in samples purchased in December.

There were significant differences in mean 4-MEI concentrations of samples purchased in California and the New York metropolitan area for Dr. Snap Regular (p = 0.03), Pepsi (p = 0.02), Diet Pepsi (p = 0.02), and Pepsi One (p<0.001). Cross-location variability diminished substantially for these same beverages measured in December 2013, reflecting declines in 4-MEI concentrations in samples purchased in New York (Table 2).

### NHANES Analyses

Table 3 shows the distribution of consumption of beverages on a typical day, by life stage. Persons reporting consumption of soft drinks overall varied considerably across the life course with 30.1% of children aged 3 to <6, 57.1% of young adults aged 16 to <21, and 34.9% of older adults aged 65 to 70 consuming any soft drinks. The percent of the population who consume each soft drink type varied as well with colas being the most popular and root beer and pepper colas being the least popular. For each soft drink type, the trend across the life course was similar with consumption highest among adolescents and young adults.

**Table 3:** Average Daily Intake in Volume (mL) among Beverage Drinkers, by Beverage and Lifestage, NHANES 2003–2010 (N = 28,710).

Beverages	% Drinkers (SE)	mL 5^th^ percentile	mL 50^th^ percentile	mL 95^th^ percentile
Any Soda (N = 14,229)				
3 to < 6^1^	30.1 (1.3)	196.5	248.1	300.7
6 to < 11	44.6 (1.2)	291.3	412.4	564.9
11 to < 16	56.5 (1.4)	444.0	581.5	753.8
16 to < 21	57.1 (1.0)	550.8	815.5	1070.3
21 to < 45	57.9 (1.1)	497.9	811.3	1143.0
45 to < 65	48.4 (1.4)	457.4	672.2	864.4
65 to 70	34.9 (1.8)	352.4	485.9	642.0
Cola				
3 to < 6	10.4 (0.8)	151.8	235.3	353.9
6 to < 11	16.8 (0.7)	265.8	352.3	464.0
11 to < 16	24.0 (1.0)	370.0	519.7	669.1
16 to < 21	25.7 (0.9)	464.0	668.1	928.0
21 to < 45	26.8 (0.9)	435.7	714.1	960.6
45 to < 65	19.2 (0.8)	389.7	615.1	807.7
65 to 70	13.3 (1.2)	360.6	459.8	682.4
Diet-Cola				
3 to < 6	2.0 (0.4)	-14.8	195.0	382.9
6 to < 11	3.5 (0.5)	214.7	294.3	402.7
11 to < 16	5.0 (0.6)	290.9	428.3	505.1
16 to < 21	4.3 (0.6)	215.0	618.9	911.4
21 to < 45	14.8 (0.7)	521.4	748.4	973.6
45 to < 65	17.1 (0.7)	396.0	723.4	1006.3
65 to 70	13.2 (1.5)	233.1	448.1	685.2
Root Beer				
3 to < 6	3.0 (0.5)	89.4	216.9	376.8
6 to < 11	4.5 (0.5)	269.4	365.8	499.5
11 to < 16	4.0 (0.4)	284.8	457,2	679.2
16 to < 21	3.1 (0.4)	390.9	702.4	940.8
21 to < 45	2.2 (0.2)	336.7	592.5	885.9
45 to < 65	1.4 (0.2)	218.6	513.2	835.9
65 to 70	2.2 (0.5)	-41.5	423.7	1055.3
Pepper Cola				
3 to < 6	2.5 (0.4)	17.4	248.3	405.0
6 to < 11	4.3 (0.6)	290.2	424.2	605.6
11 to < 16	7.1 (0.9)	399.4	506.2	601.5
16 to < 21	7.8 (0.7)	453.3	699.8	913.7
21 to < 45	5.2 (0.5)	247.4	737.5	1336.9
45 to < 65	2.3 (0.3)	371.1	631.4	846.9
65 to 70	0.9 (0.3)	-154.7	306.0	829.4
Other (non-diet) Cola				
3 to < 6	14.6 (1.0)	191.9	240.8	258.1
6 to < 11	21.9 (1.0)	274.7	361.5	463.2
11 to < 16	27.0 (1.0)	393.2	503.4	612.6
16 to < 21	26.7 (1.1)	386.6	693.2	956.8
21 to < 45	17.3 (0.5)	325.2	670.0	1005.2
45 to < 65	11.4 (0.7)	197.4	518.7	740.8
65 to 70	7.8 (0.9)	251.7	406.9	544.9

^1^Models for ages 3–20 are adjusted for sex, race, income and weight status. Models for ages 21+ are adjusted for sex, race, income, education, employment, BMI & weight loss intention.

Table 3 shows the distributions (5^th^, 50^th^ and 95^th^ percentiles) of the volume of each beverage consumed. Among drinkers of each beverage, the volume (mL) consumed differed by life stage and by soft drink type. Adolescents and young adults consumed the most of any soft drink compared to young children and older adults (e.g. 16 to <21 year olds consumed 550.1 mL—1070.3 mL of any soda compared to 457.4mL-864.4mL consumed by 45 to <65 year olds). This trend differed by beverage type. Consumption of cola and root beer among drinkers was relatively consistent across the life course (with the exception of children aged 3–11), whereas older adults who drank diet-cola drank more of it than adolescents (396.0 mL- 1006.3mL among 45 to <65 year olds compared to 290.0mL—505.1mL among 11 to <16 year olds).

### Exposure, Risk, and Burden

Table 4 presents the results of the population exposure and risk assessments for 11 beverages (Sprite was dropped from the risk analysis, per above). In general, these results followed the same pattern as the results of the 4-MEI analyses: under average exposure conditions, the highest LADDs were associated with Malta Goya (7.64x10^–3^ to 8.04x10^–3^ mg/kgBW-day), the three Pepsi beverages (6.31x10^–4^ to 4.24x10^–3^ mg/kgBW-day, depending on beverage and location), and Dr. Snap Regular (2.53x10^–4^ to 7.92x10^–4^ mg/kgBW-day, depending on location). The lowest LADDs were associated with the Coca-Cola beverages (8.02x10^–5^ to 1.01x10^–4^ mg/kgBW-day, depending on beverage and location). A similar pattern was observed under high exposure conditions. 4-MEI exposure associated with the Pepsi beverages and Dr. Snap Regular was higher in the New York area than in California.

**Table 4:** Exposure to 4-MEI and Cancer Risk and Burden, by Beverage and Location.

Brand	Beverage	Location	Average Exposure^1^			High Exposure^2^		
			LADD^3^	Risk^4^	Burden^5^	LADD^3^	Risk^4^	Burden^5^
365 Everyday Value	Dr. Snap Regular	All	5.22E-04	1.25E-05	173	2.19E-03	5.26E-05	718
365 Everyday Value	Dr. Snap Regular	CA	2.53E-04	6.07E-06	84	4.14E-04	9.95E-06	136
365 Everyday Value	Dr. Snap Regular	NY	7.92E-04	1.90E-05	263	2.19E-03	5.26E-05	718
A&W	Root Beer Regular	All	4.78E-04	1.15E-05	92	8.03E-04	1.93E-05	151
A&W	Root Beer Regular	CA	5.01E-04	1.20E-05	97	8.03E-04	1.93E-05	151
A&W	Root Beer Regular	NY	4.54E-04	1.09E-05	87	7.41E-04	1.78E-05	140
Brisk	Lemon Iced Tea	All	3.88E-04	9.31E-06	242	5.54E-04	1.33E-05	342
Brisk	Lemon Iced Tea	CA	3.84E-04	9.21E-06	239	5.54E-04	1.33E-05	342
Brisk	Lemon Iced Tea	NY	3.92E-04	9.41E-06	245	5.45E-04	1.31E-05	336
Coca Cola	Diet Coke	All	8.32E-05	2.00E-06	79	1.22E-04	2.92E-06	113
Coca Cola	Diet Coke	CA	8.02E-05	1.92E-06	76	1.17E-04	2.81E-06	109
Coca Cola	Diet Coke	NY	8.63E-05	2.07E-06	82	1.22E-04	2.92E-06	113
Coca Cola	Regular	All	9.74E-05	2.34E-06	161	1.42E-04	3.40E-06	233
Coca Cola	Regular	CA	1.01E-04	2.42E-06	167	1.42E-04	3.40E-06	233
Coca Cola	Regular	NY	9.40E-05	2.26E-06	156	1.31E-04	3.15E-06	216
Coca Cola	Zero	All	8.68E-05	2.08E-06	82	1.39E-04	3.34E-06	129
Coca Cola	Zero	CA	9.15E-05	2.20E-06	87	1.39E-04	3.34E-06	129
Coca Cola	Zero	NY	8.20E-05	1.97E-06	78	1.16E-04	2.78E-06	108
Dr. Pepper	Regular	All	2.39E-04	5.73E-06	79	4.04E-04	9.69E-06	132
Dr. Pepper	Regular	CA	2.43E-04	5.83E-06	81	4.04E-04	9.69E-06	132
Dr. Pepper	Regular	NY	2.35E-04	5.64E-06	78	3.88E-04	9.30E-06	127
Goya	Malta	All	7.89E-03	1.89E-04	4,919	1.27E-02	3.05E-04	7,832
Goya	Malta	CA	8.04E-03	1.93E-04	5,011	1.27E-02	3.05E-04	7,832
Goya	Malta	NY	7.64E-03	1.83E-04	4,764	1.15E-02	2.77E-04	7,122
Pepsi	Diet	All	1.62E-03	3.89E-05	1,532	6.44E-03	1.55E-04	5,980
Pepsi	Diet	CA	6.63E-04	1.59E-05	628	1.25E-03	3.01E-05	1,163
Pepsi	Diet	NY	2.58E-03	6.18E-05	2,437	6.44E-03	1.55E-04	5,980
Pepsi	One	All	2.09E-03	5.01E-05	1,977	8.78E-03	2.11E-04	8,155
Pepsi	One	CA	1.01E-03	2.43E-05	958	1.62E-03	3.88E-05	1,501
Pepsi	One	NY	4.24E-03	1.02E-04	4,014	8.78E-03	2.11E-04	8,155
Pepsi	Regular	All	1.53E-03	3.67E-05	2,527	6.25E-03	1.50E-04	10,285
Pepsi	Regular	CA	6.31E-04	1.52E-05	1,044	1.01E-03	2.43E-05	1,665
Pepsi	Regular	NY	2.42E-03	5.82E-05	4,009	6.25E-03	1.50E-04	10,285

^1^Average exposure assumes mean 4-MEI concentration in soft drink and median daily intake of soft drink;

^2^High exposure assumes maximum 4-MEI concentration in soft drink and 95^th^-percentile daily intake of soft drink;

^3^LADD is lifetime average daily dose (mg/kgBW/day);

^4^Risk is lifetime excess cancer risk associated with consumption of soft drink model;

^5^Burden is lifetime excess cancer cases associated with consumption of beverage by the U.S. population.

Both cancer risk and cancer burden mirrored the results of the exposure assessment: Malta Goya was associated with the highest risk (1.83x10^–4^ to 1.93x10^–4^, depending on location) and burden (4,764 to 5,011 excess lifetime cancer cases over 70 years), followed by the three Pepsi beverages (risk: 1.52x10^–5^ to 1.02x10^–4^, depending on beverage and location; burden: 628 to 4,014 cases), and Dr. Snap Regular (risk: 6.07x10^–6^ to 1.9x10^–5^, depending on location; burden: 84 to 263 cases). While the three Coca-Cola beverages posed the lowest risk (1.92x10^–6^ to 2.42x10^–6^, depending on location) and generally these beverages were associated with lower burdens (76 to 167 cases), Dr. Pepper from all locations and from California and the New York area was associated with a lower burden (78 to 81 cases) than Coca-Cola beverages from some locations. It is important to note that risks and associated burdens were driven both by varying 4-MEI concentrations in beverages and by varying consumption rates for the different beverages. Cancer risks and burdens for adults by race/ethnicity are presented in Table D in S1 File.

## Discussion

This is the first study to estimate 4-MEI concentrations in common soft drinks as well as corresponding cancer risks and burdens. Based on 4-MEI concentrations observed in beverage samples from this study, it appears that 4-MEI exposures associated with average rates of soft drink consumption pose excess cancer risks exceeding one case per 1,000,000 exposed individuals, which is a common acceptable risk goal used by some U.S. federal regulatory agencies [20–22]. Specifically, consumption of Malta Goya, Pepsi, Diet Pepsi and Pepsi One resulted in 4-MEI exposures with associated risks exceeding one excess case per 10,000 exposed individuals, suggesting that the risk can greatly exceed this threshold.

Caramel color is regulated as a color additive by the by the U.S. Food and Drug Administration (FDA)[3,23]. Despite increasing public concerns regarding risks related to 4-MEI exposure, the FDA’s action has been limited to continued assessment of the occurrence of 4-MEI in foods and beverages containing caramel color, rather than characterization of risks or regulatory action [24,25]. FDA has recently completed an examination of 400 different foods for the presence of 4-MEI, and concluded that caramel color-containing carbonated beverages contributed to approximately 25% of 4-MEI intake in the US population over age 2, accounting for more exposure than any other source [24].

Still, in statements made to the media, FDA officials have suggested that highly unrealistic soda consumption (i.e., 1,000 cans per day) would be required to achieve the 4-MEI exposures that induced cancer in toxicological studies, implying the existence of an exposure threshold for carcinogenesis [9]. Similar statements are made regularly by the beverage industry, which submitted these claims to the OEHHA when the agency set the NSRL for 4-MEI under Proposition 65 [26]. The OEHHA denied these claims on the basis of available evidence regarding the mode of carcinogenic action of 4-MEI, concluding that while little evidence supported a genotoxic mode of action for 4-MEI, the existing battery of tests was insufficient to rule out its possibility, especially in the lung. Thus, the OEHHA concluded that, in the absence of evidence for an alternative mode of action, it would default to assuming that 4-MEI acts linearly in the low-dose region of the dose-response curve, and that a threshold does not exist [14]. In this study, we followed the OEHHA’s interpretation of the data and the shape of the dose-response curve.

Our results demonstrated marked differences in 4-MEI concentrations between California (where Proposition 65 applies) and the New York area (where no similar regulation is present). With one exception (Goya Malta, where concentrations were consistently high and did not vary by time and location), 4-MEI concentrations in beverages were always considerably elevated in samples purchased in the New York area compared to those purchased in California. This difference was striking even in light of the limited number of samples tested. This supports the notion that Proposition 65 and, potentially, other state-level interventions can incentivize manufacturers to reduce foodborne chemical exposures and associated risks among consumers.

There are several notable limitations. We estimated 4-MEI exposure and associated cancer risk and burden separately for specific beverages. These estimates assume that the U.S. population drinks one beverage exclusively and that the population is not exposed to 4-MEI from additional sources. As shown here, a variety of beverages contain 4-MEI at levels of public health concern and individuals may consume more than one beverage type. Additional exposure pathways may include other beverages like beer and blended whiskey; foods like baked goods, gravies, and sauces; and secondhand smoke [12]. Another limitation was the lack of specific intake rates and consumption fractions for malt beverages and iced teas. With respect to the analyses using the NHANES data, the use of single 24-hour dietary recalls may introduce inaccuracy and bias due to underreporting, unreliability, or conversion error. Lastly, our beverage concentration estimates are based on the testing of a small number of lots from two geographic locations; despite this, in most cases, 4-MEI concentrations were relatively consistent across lots. It is possible that further differences in concentrations could be observed if samples from other geographic locations were tested.

Even considering the impact of Proposition 65 on 4-MEI concentrations in beverages, it is worth noting that the NSRL established by OEHHA corresponds to a risk of one cancer per 100,000 people exposed. Given that a sizable fraction of the U.S. population consumes these beverages, and high consumption by some persons, a substantial cancer burden may persist even if exposures are reduced to the NSRL nationally. Accordingly, federal regulation to eliminate unnecessary 4-MEI exposures may be needed. An FDA intervention, such as maximum levels for 4-MEI in beverages, could be a valuable approach to reducing excess cancer risk attributable to 4-MEI exposure in the U.S. population.

## Supporting Information

S1 File Table A. Percentage of Drinkers among the General Population and Average Daily Consumption in Volume (mL) among Beverage Drinkers for U.S. Adults Age 21–70, Overall and by Race/Ethnicity, NHANES 2003–2010 (N = 14,096)^1^. Caption: ^1^Models adjusted for sex, age, race, education, employment, income, BMI, and weight loss intention. **Table B. NHANES soft drink classifications used as bases for soda model intake rate and consumption factor estimates**. Caption: ^1^Goya Malta and Brisk Lemon Iced Tea did not rely on NHANES intake factors or consumption factors from specific soft drink classifications. Instead, they used the median and maximum IRs and the CFs of all of the NHANES categories used for the exposure assessment (including cola, diet cola, pepper cola, and root beer). **Table C. Bodyweight (kg) and Exposure Duration (years), by Lifestage**. Caption: ^1^ Body weight estimates are recommended values from the United States Environmental Protection Agency (2011) Exposure Factors Handbook, Table 8-1, available at: http://www.epa.gov/ncea/efh/pdfs/efh-complete.pdf. **Table D. Exposure to 4-MEI and Cancer Risk and Burden, by Race/Ethnicity and Beverage**. Caption: ^1^LADD is lifetime average daily dose (mg/kgBW/day) and assumes the mean beverage concentration of 4-MEI, the median beverage intake rate, and 70 years of daily consumption; ^2^Risk is lifetime excess cancer risk associated with consumption of soft drink model; ^3^CF is the fraction of the specified race/ethnicity group consuming the noted beverage; ^4^Estimates of race/ethnicity group populations are from the 2010 United States Census (http://www.census.gov/prod/cen2010/briefs/c2010br-02.pdf) ^5^Burden is lifetime excess cancer cases associated with consumption of beverage by the U.S. population.
(DOCX)
