# Supplementary material for: Caramel Color in Soft Drinks and Exposure to 4-Methylimidazole: A Quantitative Risk Assessment
Source: PLoS One. 2015 Feb 18;10(2):e0118138. doi: 10.1371/journal.pone.0118138 (PMC4333292; doi:10.1371/journal.pone.0118138)
Supplement: S1 File — Caption: 1Models adjusted for sex, age, race, education, employment, income, BMI, and weight loss intention. Table B. NHANES soft drink classifications used as bases for soda model intake rate and consumption factor estimates. Caption: 1Goya Malta and Brisk Lemon Iced Tea did not rely on NHANES intake factors or consumption factors from specific soft drink classifications. Instead, they used the median and maximum IRs and the CFs of all of the NHANES categories used for the exposure assessment (including cola, diet cola, pepper cola, and root beer). Table C. Bodyweight (kg) and Exposure Duration (years), by Lifestage. Caption: 1 Body weight estimates are recommended values from the United States Environmental Protection Agency (2011) Exposure Factors Handbook, Table 8-1, available at: http://www.epa.gov/ncea/efh/pdfs/efh-complete.pdf. Table D. Exposure to 4-MEI and Cancer Risk and Burden, by Race/Ethnicity and Beverage. Caption: 1LADD is lifetime average daily dose (mg/kgBW/day) and assumes the mean beverage concentration of 4-MEI, the median beverage intake rate, and 70 years of daily consumption; 2Risk is lifetime excess cancer risk associated with consumption of soft drink model; 3CF is the fraction of the specified race/ethnicity group consuming the noted beverage; 4Estimates of race/ethnicity group populations are from the 2010 United States Census (http://www.census.gov/prod/cen2010/briefs/c2010br-02.pdf) 5Burden is lifetime excess cancer cases associated with consumption of beverage by the U.S. population. (DOCX) [file pone.0118138.s001.docx]

**Caramel Color and Exposure to 4-Methylimidazole in Soft Drinks: A Cancer Risk Assessment**

**SUPPORTING INFORMATION**

**Additional Analytical Details and Results of Quality Control Analyses**

The analytical method to determine the concentration of 4-MEI in beverage using LC/MS/MS was validated in the commercial lab. System suitability, reproducibility, response function, calibration range, measurement limits, precision and accuracy, ruggedness, extraction efficiency, selectivity, dilution integrity, and analyte stability were evaluated to be satisfactory for the sample matrix. The limit of detection (LOD) for 4-MeI was 0.36 ng/mL and the lower limit of quantitation (LLOQ) was 1 ng/mL. The precision of the method was evaluated at three different levels (low: 5 ng/mL, medium: 50ng/mL and high 250nmg/mL). The precision of the method was excellent, as indicated by coefficient of variation (CV) less than 7% at all three levels. In addition to all standard samples used for quality control during batch analysis, we included 7 blind duplicate samples that were from the same lot and batch number during the analysis of beverage samples. The laboratory was unaware that these selected samples were duplicates. The results showed very good agreement between the duplicate samples based on simple linear regression (r-square >0.99 and slope close to 1). Accuracy of the method was evaluated using spiked samples that were scattered in between the unknown samples. Results showed that the observed concentrations for 4-MeI were 93-101% of expected concentrations.

To cross-check the results of sample analyses from the first laboratory, 12 of the original samples tested in the first contract laboratory with concentration of 4-MEI from the lowest to the highest were sent to a second contract laboratory for blinded 4-MEI analysis using the same LC/MS/MS method but validated independently. The inter-laboratory variation ranged from 2.9 – 12.8% (mean: 6.7%), indicating very good agreement between these laboratories.

# Table A. Percentage of Drinkers among the General Population and Average Daily Consumption in Volume (mL) among Beverage Drinkers for U.S. Adults Age 21-70, Overall and by Race/Ethnicity, NHANES 2003-2010 (N=14,096)^1^.

| **Beverages** | **% drinkers (SE)** | **mL 5^th^ percentile** | **mL 50^th^ percentile** | **mL 95^th^ percentile** |
| --- | --- | --- | --- | --- |
| Any Soda (N = 7,359) |  |  |  |  |
| Overall | 52.7 (1.1) | 393.2 | 731.8 | 1026.7 |
| White, non-Hispanic | 54.0 (1.4) | 498.2 | 852.9 | 1179.5 |
| Black, non-Hispanic | 46.6 (1.1) | 496.7 | 700.3 | 856.9 |
| Hispanic | 54.3 (1.5) | 423.2 | 635.9 | 837.4 |
| Cola |  |  |  |  |
| Overall | 23.0 (0.7) | 389.9 | 651.1 | 903.7 |
| White, non-Hispanic | 22.3 (0.9) | 450.9 | 746.4 | 1063.1 |
| Black, non-Hispanic | 20.2 (1.1) | 501.3 | 661.8 | 807.4 |
| Hispanic | 29.7 (1.9) | 360.9 | 599.1 | 796.8 |
| Diet Cola |  |  |  |  |
| Overall | 15.7 (0.5) | 408.7 | 703.7 | 970.7 |
| White, non-Hispanic | 18.2 (0.7) | 547.0 | 829.3 | 1051.7 |
| Black, non-Hispanic | 4.3 (0.4) | 292.9 | 546.7 | 780.3 |
| Hispanic | 10.5 (0.7) | 328.4 | 625.0 | 933.3 |
| Root-Beer |  |  |  |  |
| Overall | 1.9 (0.2) | 300.5 | 530.5 | 764.3 |
| White, non-Hispanic | 2.2 (0.2) | 287.3 | 550.9 | 834.5 |
| Black, non-Hispanic | 1.5 (0.3) | 236.1 | 511.1 | 840.1 |
| Hispanic | 1.1 (0.2) | 157.2 | 471.5 | 863.9 |
| Pepper Cola |  |  |  |  |
| Overall | 3.9 (0.4) | 166.4 | 645.7 | 1084.2 |
| White, non-Hispanic | 4.4 (0.5) | 240.3 | 782.7 | 1305.3 |
| Black, non-Hispanic | 2.8 (0.4) | 186.4 | 586.9 | 848.9 |
| Hispanic | 2.9 (0.4) | 198.4 | 553.3 | 877.6 |
| Other (non-diet) Cola |  |  |  |  |
| Overall | 14.5 (0.4) | 243.2 | 587.5 | 902.0 |
| White, non-Hispanic | 12.9 (0.5) | 334.0 | 703.3 | 1038.4 |
| Black, non-Hispanic | 23.3 (0.8) | 360.9 | 581.7 | 773.3 |
| Hispanic | 15.3 (0.7) | 249.3 | 367.9 | 670.3 |

^1^Models adjusted for sex, age, race, education, employment, income, BMI, and weight loss intention.

# Table B. NHANES soft drink classifications used as bases for soda model intake rate and consumption factor estimates.

| **NHANES soft drink classification** | **Soda model(s)^1^** |
| --- | --- |
| Cola | Coca-Cola (regular), Pepsi (regular) |
| Diet-Cola | Coca-Cola Zero, Diet Coke, Diet Pepsi, Pepsi One |
| Root Beer | A&W Root Beer |
| Pepper Cola | Dr. Pepper, Dr. Snap (regular) |

^1^Goya Malta and Brisk Lemon Iced Tea did not rely on NHANES intake factors or consumption factors from specific soft drink classifications. Instead, they used the median and maximum IRs and the CFs of all of the NHANES categories used for the exposure assessment (including cola, diet cola, pepper cola, and root beer).

# Table C. Bodyweight (kg) and Exposure Duration (years), by Lifestage.

| **Lifestage (years of age)** | **Bodyweight (kg)** | **Exposure Duration (years)** |
| --- | --- | --- |
| 3 to < 6 | 18.6^1^ | 3 |
| 6 to < 11 | 31.8 | 5 |
| 11 to < 16 | 56.8 | 5 |
| 16 to < 21 | 71.6 | 5 |
| 21 to < 45 | 80 | 24 |
| 45 to < 65 | 80 | 20 |
| 65 to 70 | 80 | 5 |

^1^ Body weight estimates are recommended values from the United States Environmental Protection Agency (2011) Exposure Factors Handbook, Table 8-1, available at: <http://www.epa.gov/ncea/efh/pdfs/efh-complete.pdf>.

**Table D. Exposure to 4-MEI and Cancer Risk and Burden, by Race/Ethnicity and Beverage.**

| **Race/ethnicity** | **Brand** | **Beverage** | **LADD^1^** | **Risk^2^** | **CF^3^** | **Population^4^** | **Burden^5^** |
| --- | --- | --- | --- | --- | --- | --- | --- |
| Overall | 365 Everyday Value | Dr. Snap Regular | 4.93E-04 | 1.18E-05 | 0.039 | 316,159,818 | 146 |
|  | A&W | Root Beer Regular | 4.31E-04 | 1.03E-05 | 0.019 | 316,159,818 | 62 |
|  | Brisk | Lemon Iced Tea | 3.77E-04 | 9.05E-06 | 0.098 | 316,159,818 | 280 |
|  | Coca Cola | Diet Coke | 8.66E-05 | 2.08E-06 | 0.157 | 316,159,818 | 103 |
|  | Coca Cola | Regular | 9.52E-05 | 2.29E-06 | 0.23 | 316,159,818 | 166 |
|  | Coca Cola | Zero | 9.02E-05 | 2.17E-06 | 0.157 | 316,159,818 | 108 |
|  | Dr. Pepper | Regular | 2.26E-04 | 5.41E-06 | 0.039 | 316,159,818 | 67 |
|  | Goya | Malta | 7.66E-03 | 1.84E-04 | 0.098 | 316,159,818 | 5698 |
|  | Pepsi | Diet | 1.68E-03 | 4.04E-05 | 0.157 | 316,159,818 | 2006 |
|  | Pepsi | One | 2.17E-03 | 5.21E-05 | 0.157 | 316,159,818 | 2588 |
|  | Pepsi | Regular | 1.49E-03 | 3.59E-05 | 0.23 | 316,159,818 | 2607 |
| White, Non-Hispanic | 365 Everyday Value | Dr. Snap Regular | 5.98E-04 | 1.43E-05 | 0.044 | 196,817,552 | 124 |
|  | A&W | Root Beer Regular | 4.48E-04 | 1.07E-05 | 0.022 | 196,817,552 | 47 |
|  | Brisk | Lemon Iced Tea | 4.44E-04 | 1.07E-05 | 0.113 | 196,817,552 | 237 |
|  | Coca Cola | Diet Coke | 1.02E-04 | 2.45E-06 | 0.182 | 196,817,552 | 88 |
|  | Coca Cola | Regular | 1.09E-04 | 2.62E-06 | 0.223 | 196,817,552 | 115 |
|  | Coca Cola | Zero | 1.06E-04 | 2.55E-06 | 0.182 | 196,817,552 | 91 |
|  | Dr. Pepper | Regular | 2.73E-04 | 6.56E-06 | 0.044 | 196,817,552 | 57 |
|  | Goya | Malta | 9.04E-03 | 2.17E-04 | 0.113 | 196,817,552 | 4823 |
|  | Pepsi | Diet | 1.98E-03 | 4.76E-05 | 0.182 | 196,817,552 | 1706 |
|  | Pepsi | One | 2.56E-03 | 6.14E-05 | 0.182 | 196,817,552 | 2201 |
|  | Pepsi | Regular | 1.71E-03 | 4.11E-05 | 0.223 | 196,817,552 | 1804 |
| Black, Non-Hispanic | 365 Everyday Value | Dr. Snap Regular | 4.48E-04 | 1.08E-05 | 0.028 | 37,685,848 | 11 |
|  | A&W | Root Beer Regular | 4.15E-04 | 9.97E-06 | 0.015 | 37,685,848 | 6 |
|  | Brisk | Lemon Iced Tea | 3.29E-04 | 7.91E-06 | 0.0355 | 37,685,848 | 11 |
|  | Coca Cola | Diet Coke | 6.72E-05 | 1.61E-06 | 0.043 | 37,685,848 | 3 |
|  | Coca Cola | Regular | 9.68E-05 | 2.32E-06 | 0.202 | 37,685,848 | 18 |
|  | Coca Cola | Zero | 7.01E-05 | 1.68E-06 | 0.043 | 37,685,848 | 3 |
|  | Dr. Pepper | Regular | 2.05E-04 | 4.92E-06 | 0.028 | 37,685,848 | 5 |
|  | Goya | Malta | 6.70E-03 | 1.61E-04 | 0.0355 | 37,685,848 | 215 |
|  | Pepsi | Diet | 1.31E-03 | 3.14E-05 | 0.043 | 37,685,848 | 51 |
|  | Pepsi | One | 1.69E-03 | 4.05E-05 | 0.043 | 37,685,848 | 66 |
|  | Pepsi | Regular | 1.52E-03 | 3.64E-05 | 0.202 | 37,685,848 | 277 |
| Hispanic | 365 Everyday Value | Dr. Snap Regular | 4.23E-04 | 1.01E-05 | 0.029 | 47,435,002 | 14 |
|  | A&W | Root Beer Regular | 3.83E-04 | 9.19E-06 | 0.011 | 47,435,002 | 5 |
|  | Brisk | Lemon Iced Tea | 3.35E-04 | 8.04E-06 | 0.067 | 47,435,002 | 26 |
|  | Coca Cola | Diet Coke | 7.69E-05 | 1.85E-06 | 0.105 | 47,435,002 | 9 |
|  | Coca Cola | Regular | 8.76E-05 | 2.10E-06 | 0.297 | 47,435,002 | 30 |
|  | Coca Cola | Zero | 8.02E-05 | 1.92E-06 | 0.105 | 47,435,002 | 10 |
|  | Dr. Pepper | Regular | 1.93E-04 | 4.64E-06 | 0.029 | 47,435,002 | 6 |
|  | Goya | Malta | 6.81E-03 | 1.63E-04 | 0.067 | 47,435,002 | 519 |
|  | Pepsi | Diet | 1.50E-03 | 3.59E-05 | 0.105 | 47,435,002 | 179 |
|  | Pepsi | One | 1.93E-03 | 4.63E-05 | 0.105 | 47,435,002 | 231 |
|  | Pepsi | Regular | 1.37E-03 | 3.30E-05 | 0.297 | 47,435,002 | 465 |

^1^LADD is lifetime average daily dose (mg/kgBW/day) and assumes the mean beverage concentration of 4-MEI, the median beverage intake rate, and 70 years of daily consumption; ^2^Risk is lifetime excess cancer risk associated with consumption of soft drink model; ^3^CF is the fraction of the specified race/ethnicity group consuming the noted beverage; ^4^Estimates of race/ethnicity group populations are from the 2010 United States Census (http://www.census.gov/prod/cen2010/briefs/c2010br-02.pdf) ^5^Burden is lifetime excess cancer cases associated with consumption of beverage by the U.S. population.
